# The role of ANS acuity and numeracy for the calibration and the coherence of subjective probability judgments

**DOI:** 10.3389/fpsyg.2014.00851

**Published:** 2014-08-05

**Authors:** Anders Winman, Peter Juslin, Marcus Lindskog, Håkan Nilsson, Neda Kerimi

**Affiliations:** Department of Psychology, Uppsala UniversityUppsala, Sweden

**Keywords:** subjective probability judgments, calibration, coherence, numeracy, approximate number system acuity

## Abstract

The purpose of the study was to investigate how numeracy and acuity of the approximate number system (ANS) relate to the calibration and coherence of probability judgments. Based on the literature on number cognition, a first hypothesis was that those with lower numeracy would maintain a less linear use of the probability scale, contributing to overconfidence and nonlinear calibration curves. A second hypothesis was that also poorer acuity of the ANS would be associated with overconfidence and non-linearity. A third hypothesis, in line with dual-systems theory (e.g., Kahneman and Frederick, 2002) was that people higher in numeracy should have better access to the normative probability rules, allowing them to decrease the rate of conjunction fallacies. Data from 213 participants sampled from the Swedish population showed that: (i) in line with the first hypothesis, overconfidence and the linearity of the calibration curves were related to numeracy, where people higher in numeracy were well calibrated with zero overconfidence. (ii) ANS was not associated with overconfidence and non-linearity, disconfirming the second hypothesis. (iii) The rate of conjunction fallacies was slightly, but to a statistically significant degree decreased by numeracy, but still high at all numeracy levels. An unexpected finding was that participants with better ANS acuity gave more realistic estimates of their performance relative to others.

## Introduction

Although typical judgment and decision making tasks have always required participants to process numerical information, major interest in how the ability to process numerical information affect the ability to make judgments and decisions was sparked only recently (see e.g., Peters et al., 2008). This interest has focused on the concept of numeracy, typically defined as the ability to understand and process numerical information (Reyna et al., 2009). The research has for example suggested that people lower on numeracy are more sensitive to framing effects in decision making (Peters et al., 2006; Reyna et al., 2009), make less accurate risk estimates (Black et al., 1995), and ignore sample size information to a larger extent (Obrecht et al., 2009).

How numeracy affects the accuracy of probability judgments has received less attention (but see, Dieckmann et al., 2009; Lipkus et al., 2010). With a *correspondence* criterion, the accuracy of probability judgments is evaluated by comparison to an independently defined objective criterion, like the relative frequency with which the event occurs. With a *coherence criterion*, probability judgments are evaluated by the extent to which they obey the rules of probability theory. In other words, the extent to which they are internally consistent (Hammond, 1996; Nilsson et al., 2013). The two studies that we know of that investigate the effect of numeracy on coherence suggest divergent conclusions. Wedell (2011) reported no significant relationship between numeracy and rate of conjunction errors while Liberali et al. (2012) reported that people with high numeracy made fewer conjunction (and disjunction) errors. We know of no studies that investigate the effect of numeracy on the correspondence of probability judgments. Regardless of the criterion, previous research documents extensive individual differences in the ability to make accurate probability judgments and the studies often suggest divergent conclusions.

In this study we investigate the effect of numeracy on both the coherence and the correspondence of probability judgments. We include two important features to obtain high generalizability. First, the way in which the task material is selected is known to affect the normative evaluation of probability judgments (Gigerenzer et al., 1991; Juslin, 1993; Björkman, 1994) and therefore influences who is considered a “good” or a “poor” probability assessor. The probability estimation tasks were therefore designed to meet the criterion of representative design (Brunswik, 1956; Dhami et al., 2004) to allow for conclusions that are not driven by idiosyncrasies in the stimulus material and which generalize to the participants' natural environment. Second, our participants were recruited from randomly sampled data lists from the population of Uppsala. While some previous studies have used large samples of participants (e.g., Wedell, 2011), to our knowledge no study has tried to incorporate a variability that is approximately representative of the general population. In the following, we first consider mechanisms by which individual differences in numerical ability can affect the ability to assess and integrate probabilities. Thereafter, we report data on how numerical abilities relate to the correspondence and the coherence of probability judgments.

## Correspondence and coherence of probability judgments

### Calibration (correspondence)

Calibration refers to the degree to which subjective probabilities correspond to relative frequencies (Lichtenstein et al., 1982). For example, across a set of questions, for which a judge assesses the probability of being correct to 0.6, 60% should be correct. The *over/underconfidence* bias is measured by the mean subjective probability minus the proportion correct (relative frequency). A positive score is overconfidence, with too high confidence, whereas a negative score indicates underconfidence (Lichtenstein and Fischhoff, 1977). People are often reported to be overconfident. The “overconfidence phenomenon” has been described as a pervasive cognitive bias (Lichtenstein et al., 1982; Arkes, 1991; Baron, 1994) and as “ubiquitous” (West and Stanovich, 1997). De Bondt and Thaler (1995) argued that “Perhaps the most robust finding in the psychology of judgment is that people are overconfident.” These conclusions diverge from the “ecological arguments” (Gigerenzer et al., 1991; Juslin, 1993; Björkman, 1994) suggesting that overconfidence is, at least in part, driven by over-selection of tricky and unusual items that are exceptions to the statistical regularities that obtain in the real-world. When tested on item samples where the content is randomly sampled from a natural environment, overconfidence is accordingly often reduced or eliminated (see Juslin et al., 2000; see Moore and Healy, 2008 and Koriat, 2012, for discussions of other causes of overconfidence).

There are a number of studies of individual differences in overconfidence, as measured by subjective probability calibration. West and Stanovich (1997) reported a significant, but low, correlation between overconfidence in a general knowledge task, and in a motor performance task. Bornstein and Zickafoose (1999) likewise found correlations between performance in a general knowledge task and performance in an eyewitness memory task. In Stanovich and West (1998a) overconfidence was positively correlated with the false consensus effect and negatively correlated with measures of cognitive ability. Klayman et al. (1999) found stable individual differences, but substantial variation in over/underconfidence depending on the question type. Jonsson and Allwood (2003) found some individual stability over time, but substantial individual differences across task domains, and no correlation between need for cognition and over/underconfidence. As noted, some inconsistencies might be attributed to lack of control in the stimulus material. A person that is overconfident with selected items may well be perfectly calibrated with a representative sample of items. We know of no study on individual differences in calibration with representative general knowledge items.

In the tasks above, overconfidence means that participants overestimate their own ability relative to an absolute norm. Another version of overconfidence is in terms of “overplacement,” or the “better-than-average effect” (Merkle and Weber, 2011), whereby participants overestimate how well they perform in relation to others. In a typical setup, people are asked to judge whether they are above or below average in a certain domain. In other studies, participants specify the percentile of a distribution that they believe themselves to belong to in regard to a particular skill. The typical finding is that people rate themselves as being better than they actually are relative to others. For example, most people believe that they are a better driver than the average driver (Svenson, 1981). Merkle and Weber (2011) concluded that results within this paradigm showed “true overconfidence” appearing as “a consequence of a psychological bias.” One criticism of this task is that people may interpret the often vaguely defined skill differently. If people rate different aspects of car driving, they may actually be better than average when this is taken into account. The effect may also stem from the use of sub populations as participants. If students are used as participants, it is possible that they actually perform at higher levels than the general population at a particular skill (e.g., on an IQ test). In spite of this criticism, we are not aware of a single study that has used a representative sample of participants and instructions with an unambiguous and exact definition of both the task and the comparison population.

### The conjunction fallacy (coherence)

When no “objective” probability exists, probability estimates can be evaluated by the extent to which they *cohere* with the laws of probability. Kahneman and Tversky (1982) presented participants with a description of Linda, a stereotypical feminist, and asked them whether she was more likely to be a bank-teller (*A*) or a bank-teller and a feminist (*A*∩ B). Almost 90% of the participants committed the conjunction fallacy, by estimating that she was more likely to be a feminist bank-teller (which is logically impossible given that *A*∩ B is a subset of *A*). Since then, numerous studies have shown that the fallacy is robustly observed in a range of different populations (e.g., Davidson, 1995; Adam and Reyna, 2005) and different tasks (e.g., Zizzo, 2003; Nilsson, 2008; Nilsson et al., 2009). In contrast to overconfidence, the conjunction fallacy does not appear to be reduced by use of representative design (Nilsson et al., 2009).

Conjunction fallacies seem to be explained by two mechanisms. First, people often combine the constituent probabilities as a configural weighted average (Gavanski and Roskos-Ewoldsen, 1991; Nilsson, 2008; Nilsson et al., 2009; Jenny et al., 2014). Second, the rate of conjunction fallacies is mediated by the inductive confirmation for the conjunction (Tentori et al., 2013). The effect is thus affected by the believability of the conjunctive event (Kahneman and Tversky, 1982; Tentori et al., 2013) with the rate of fallacies dropping if the conjunctions include contradictory conjuncts such as “Alan is bored with music” and “Alan plays Jazz for a hobby.” It is moreover often assumed that the responses are negotiated by two cognitive systems. An intuitive system that tends to produce conjunction fallacies that is (imperfectly) monitored by an analytic system with some insight about the rules of probability theory (Kahneman and Frederick, 2002; Peters et al., 2006; Evans, 2008), where the latter is partially tapped by numeracy (Liberali et al., 2012).

Little attention has been given to individual differences. However, in some studies high cognitive ability is correlated with lower rates of fallacies (e.g., SAT-scores, Stanovich and West, 1998b; Feeney et al., 2007). In other studies there is no such relationship (Stanovich and West, 1998b; Feeney et al., 2007; Wedell, 2011). Wedell (2011, p. 157) for example, concluded that “*Need for cognition and numeracy were only minimally related to reasoning about conjunctions*.” while Liberali et al. (2012) reported a correlation between numeracy and the rate of conjunction errors. One account of the different results could be that previous studies have used different measures of numeracy and often measures with poor psychometric properties (see, e.g., Cokely et al., 2012).

## Number perception and abilities in humans

In the following section we give an overview of two basic numerical abilities. The first, an innate ability for the understanding of numerosities, is primarily concerned with the evaluation of non-symbolic magnitudes and stems from an innate approximate number system. The second, a culturally acquired ability for understanding numerical information, is concerned with the manipulation and understanding of exact numbers.

Human adults, children, and non-human animals can represent numerical magnitudes without use of symbols (Feigenson et al., 2004). The underlying system, known as the Approximate Number System (ANS), represents numbers and magnitudes in an analog and approximate fashion, with increasingly imprecise representations as numerosity increases (Gallistel and Gelman, 1992, 2000). The increasing imprecision makes comparisons between small magnitudes (e.g., 10 and 20) easier than comparisons between large magnitudes (e.g., 1010 and 1020). Numerosities are thus scaled in a nonlinear fashion. The acuity of the ANS is often conceptualized as the smallest change in numerosity, as quantified by a Weber fraction (*w*) (Pica et al., 2004; Halberda and Feigenson, 2008; Halberda et al., 2008, 2012; Tokita and Ishiguchi, 2010), which can be detected. Several studies document considerable individual variation in ANS acuity (Pica et al., 2004; Halberda and Feigenson, 2008; Halberda et al., 2008; Tokita and Ishiguchi, 2012; Lindskog et al., 2013). A first numerical ability thus involves a nonlinear, ordinal appreciation for magnitudes.

Modern society increasingly requires use of number information on the exact linear number scale. The ability to understand and process numeric information, summarized in the concept of *Numeracy* (Paulos, 1988; in analogy with literacy), has recently attracted interest in research on decision-making (Reyna and Brainerd, 2007; Peters and Levin, 2008; Peters et al., 2008; Reyna et al., 2009). Although no consensus can be found on how numeracy should be defined (see Reyna et al., 2009 for a review) it often refers to an understanding of, and an ability to process, numerical concepts, particularly to comprehend risk and to transform probabilities (Lipkus et al., 2001). A remarkable proportion of even highly educated people apparently lack knowledge of basic numeric concepts (Lipkus et al., 2001). A second numerical ability thus refers to culturally acquired knowledge of the linear number scale and the algebraic operations performed on it, including an understanding of the notion of “probability” and at least some rudimentary knowledge of the probability rules.

While ANS is mostly concerned with non-symbolic numerical magnitudes and numeracy refers to the manipulation of exact numbers, several pieces of evidence suggest a link between the two. First, previous studies suggest a relationship between ANS acuity and math achievement that holds also when controlling for other cognitive abilities (Halberda et al., 2008). Second, it has been indicated that formal mathematics education might influence the acuity of the ANS (Nys et al., 2013). Finally, when exact numbers are encoded on the internal number line, the logarithmic compression often becomes evident. For example, the distance effect (Dehaene et al., 1990) and the size effect (Barth et al., 2003) indicate an increasing inaccuracy of representations for larger numbers. Tasks in which participants estimate the spatial position of numbers on a line indicate that children show a non-linear representation of numbers (Siegler and Opfer, 2003), an effect that declines with age as people become “more linear.” This suggests that the original non-linear representation coexists with, rather than is replaced by, the linear representation acquired later (Siegler and Opfer, 2003; Opfer and DeVries, 2008). The latter develops from experience with numbers and mathematical education, which is in part indexed by the person's numeracy. A more general notion of number perception holds that numerical quantities in a diversity of formats are represented by a common “number sense” (Dehaene, 1992) that maps quantities onto a common internal number line (Dehaene and Cohen, 1995). For example, the Arabic digit (6), six squares, or six tones (all representing the quantity *six*) are all mentally represented by the same position on the internal number line. The accuracy of this mapping may moreover be related to judgments and decisions. Schley and Peters (2014), for example, found that the extent to which people are linear on the above described number line task (Siegler and Opfer, 2003) is related to the shape of their value function.

## Number perception and probability

While numerical abilities have mainly been investigated in the context of whole numbers, there is an increasing interest in how people represent fractions or proportions (see Jacob et al., 2012; Siegler et al., 2013), which is obviously connected to the ability to represent and understand probability. While whole numbers have a lower bound at zero and no upper bound, proportions (probabilities) have both a lower bound (0) and a higher bound (1). With whole numbers the standard finding is that people discriminate better between low numbers that are close to the natural bound (0) than between high numbers (e.g., the difference 10–20 feels larger than the difference 1010–1020), often captured by assuming that the internal number line is logarithmically compressed.

**Figure 1:**
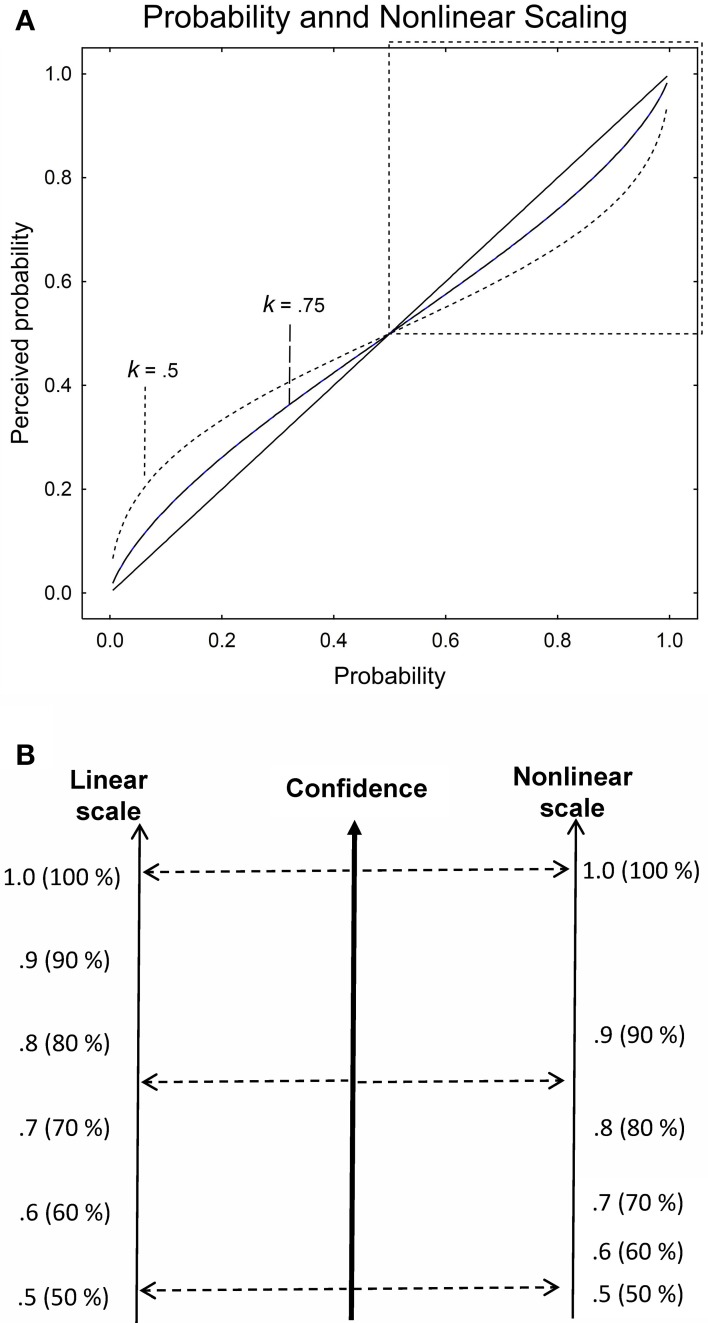
**(A)** Linear and nonlinear perception of proportions (probability). The solid identity line represents a perfectly linear representation of proportion (*k* = 1), whereas the non-linear functions are two examples of non-linear representations (*k* = 0.75 and 0.5). The upper right region of the graph delineated by a square is the region of interest in half-range probability judgment. **(B)** Schematic example of the direct and proportional mapping from an internal confidence variable to a linear and a nonlinear perception of the confidence scale in a half-range task.

The corresponding finding with proportions is that the perceived proportions^1^ are non-linear functions of the objective proportions. There is often better discrimination between the proportions close to 0 and 1 than between intermediate proportions. This is perhaps most famously captured by the decision weighting function in prospect theory (Kahneman and Tversky, 1979; Tversky and Kahneman, 1992). People find the difference between stated proportions 0.99 and 1 “larger” and easier to discriminate than the difference between stated proportions 0.50 and 0.51. A recent review (Zhang and Maloney, 2012) documents that the nonlinearity introduced in the perception of proportions (probabilities) is often well captured by a logit function. If we express perceived proportion *r* as a function of stated proportion *p* we have,

(1) $$r\left(p\right)=\frac{e^{k\;*\;ln}(p/(1-p))}{e^{k\;*\;ln}(p/(1-p))+1^{\prime }}$$

where *k* is parameter that determines the non-linearity in the perception of the proportions^2^. A person with *k* = 1 is able to maintain a perfectly linear representation of the stated proportion. This person weights the difference between 0.50 and 0.51 just as much as the difference between 0.99 and 1 in a judgment or a decision. Most people are, however, likely to have a slightly nonlinear representation of the proportions, with sharper discrimination between proportions close to 0 and to 1 (see Figure 1A for the examples of two persons with *k* = 0.75 and *k* = 0.5 respectively, as well as a person with *k* = 1, the identity line).

In the study reported in this article we rely on a half-range probability scale where participants first decide on one of two choice alternatives (True or False) and then make a confidence assessment on a scale from 0.5 to 1 in steps of 0.1. Figure 1B provides a schematic illustration of the effect produced if the internal confidence variable is mapped directly and proportionally to a linear and to a nonlinear scale. For the same confidence, a person with a nonlinear perception of the stated probability scale (*k* < 1) will produce larger values on the probability scale in the low range than a person with a linear perception of the probability scale. In other words, perceiving little difference between the lower stated probabilities on the confidence scale (0.5, 0.6, 0.7), a person with *k* < 1 will “climb” more rapidly toward intermediate confidence levels, introducing nonlinearity and overconfidence in the calibration curve. The effect is that overconfidence is introduced by the nonlinear perception of the probability scale and the calibration curve, which plots the proportions correct against the stated probability, will become curvilinear. Assuming a judge that is perfectly calibrated with a linear scale (*k* = 1), the calibration curves for *k* < 1 will look like the functions in the upper right part of Figure 1A but with confidence on the *x*-axis and proportion correct on the *y*-axis.

## Purpose of the study and predictions

In this study, we will submit a sample of participants that is approximately representative of the population to two probability assessment tasks that epitomize classical criteria of correspondence and coherence, respectively: the calibration of subjective probabilities and the obedience of the conjunction rule. The literature on numerical cognition, in conjunction with the literature on the accuracy of probability judgments, suggests that the two numerical abilities outlined above might independently affect the extent to which probability judgments adhere to correspondence and coherence criteria.

First, as we have seen, there is evidence that the representation of symbolic number is often distorted by the intrusion of an underlying nonlinear representation (e.g., Siegler and Opfer, 2003; Schley and Peters, 2014) with poorer discrimination for the numeric magnitudes that are distant from the scale bounds. *Hypothesis one* was based on the assumption that people lower in numeracy are less able to maintain a linear number representation in their perception and use of the probability scale. We accordingly expected them to be more overconfident with a more curvilinear calibration curve. Previous research indicates that judgments can be influenced by ANS acuity (Peters et al., 2008). However, the study by Peters et al. (2008) used a measure of ANS acuity (numeric distance effect) that has been challenged for its validity (Lindskog et al., 2013; Chen and Li, 2014; Inglis and Gilmore, 2014). We consequently used a measure of ANS acuity with better validity (Halberda et al., 2008; Lindskog et al., 2013). To the extent that ANS acuity is expressed in the nonlinearity of the calibration curve, *the second hypothesis* was that overconfidence is expected to be negatively related to ANS acuity. In regard to coherence, the *third hypothesis* was that people of higher numeracy, that are more versed in the culturally acquired knowledge of arithmetic operations, should be more likely to adhere to the extension rule of probability theory and commit fewer conjunction errors. By contrast, people of lower numeracy should be more likely to use the default operation of taking a configural weighted average of the conjunct probabilities (Nilsson et al., 2009), producing many conjunction errors.

In the following study we used a composite measure of numeracy consisting of an aggregate of previously used tests with known psychometric characteristics^3^. ANS acuity was measured with a standard numerosity dot comparison test with brief exposure (Halberda et al., 2008). To control for the effects of general cognitive ability the participants carried out a subset of Raven's progressive matrices. The participants also responded to single and conjunctive statements to assess their general knowledge calibration and their susceptibility to conjunction errors, allowing us to assess both the correspondence and the coherence of the judgments as a function of the participants' numeracy and ANS acuity.

## Methods

### Participants

A random sample of 2000 inhabitants of Uppsala, with the criteria of obtaining an equal gender distribution, participants being between 20 and 60 years, and living a maximum of 20 km from the town center, was ordered from Statistics Sweden (a government agency). Because Uppsala is a university town, student dominated areas were excluded. The individuals were contacted by post. Three hundred and thirty two responded to the letters. Out of these, 213 participated in the study sessions (age: *M* = 39, *SD* = 12). Sixty-two percent were women and 38% were men. Self-selection resulted in an overrepresentation of women. The sample was otherwise representative of the Swedish population (population statistics from 2012 are valid for individuals between 16 and 65 years and were obtained from Statistics Sweden). Thirty-four percent, 40, 21, and 5% reported elementary school, high school, university and other, respectively, as highest level of education (corresponding percentages in the total Swedish population are 35.3, 41.3, and 23.4%, respectively, for elementary school, high school, and university). Eighty-six percent were born in Sweden (corresponding percentages in the total Swedish population is 81%). The median monthly income was 25,000 SEK (corresponding number in the total Swedish population is 25,400 SEK). In addition to the tasks reported here, participants carried out a large battery of tasks over two laboratory sessions and one online session^4^. For compensation, participants could choose between a gift certificate with a value of 1000 SEK or donating the same amount to an optional charity.

### Materials and procedure

#### Subjective probability tasks

The general knowledge task from Nilsson et al. (2009) was used to elicit subjective probability estimates and confidence ratings. While the task is similar to the tasks typically used in the literature on calibration, it deviates from the tasks typically used in the literature on the conjunction fallacy where most tasks are of the “Linda-type” described above. There were three main reasons for relying on this task. First, because the task involves factual statements that can be either true or false it enables analyses of both the coherence and the correspondence of the probability estimates. Second, because stimuli are randomly sampled from a real world environment it meets the criterion of representative design (Brunswik, 1956), described above. Third, the large number of estimates and ratings provided by each participant ensures high statistical power. Estimates and ratings were given to single statements and conjunctive statements.

***Single factual statements***. In the general knowledge task the single statements consisted of 100 propositions about geographical facts that participants were to judge to be true or false, e.g., “France has a larger area than Costa Rica.” A base rate of 50% propositions were true. Six different topics were used [Area/larger, population (country)/larger, population (capital)/larger, latitude (capital)/farther north, population density/more dense, and life expectancy/higher]. The items were randomly sampled from a database of world countries with the restriction of an even number of items from each topic. After each question the participant gave a confidence rating from 50 (guessing) to 100% (absolutely certain) in the accuracy of their answer. A written instruction presented a frequentist interpretation of the probability scale to participants (i.e., “For all answers where you have given a 70% confidence rating 70% are expected to be correct in the long run.”). All measures of overconfidence, miscalibration, estimation, and placement (defined below) were calculated on ratings from the general knowledge task in this single component condition.

***Conjunctive factual statements***. The propositions from the single component statements of the general knowledge task described above were randomly divided into 50 pairs (hence, each component was included in one pair). Sampling was restricted so that each pair included two components with the same target variable.

Each pair was converted into a conjunction. For example, the following two components: “France has more inhabitants than Peru” and “Sweden has more inhabitants than Estonia” would occur with the conjunction “France has more inhabitants than Peru and Sweden has more inhabitants than Estonia.” Thus, conjunctive statements consisted of the same pairs of components that were identical but for the word “and” inserted between the components. Participants were told that they would be shown statements about geographical facts. They were provided with an example and told that for a conjunction to be true it is necessary that both component propositions are true. Participants were informed that the task was to judge whether the statement was true or false and to rate the confidence in this answer on the same scale as described above.

***Numeracy***. Participants completed (in the following order) the *Expanded Numeracy test* (Lipkus et al., 2001), the *Berlin Advanced Numeracy Test* (Cokely et al., 2012), and the *Subjective Numeracy Scale* (Fagerlin et al., 2009). A composite measure of numeracy was calculated by averaging these standardized measures. The three tests each focuses on a fairly narrow part of the multifaceted concept of numeracy. In an attempt to catch the effects of numeracy in general, rather than the effects of a subset of its sub dimensions, the composite measure was calculated by averaging across the three, significantly inter-correlated [Expanded Numeracy test—Berlin Advanced Numeracy Test: *r*_(211)_ = 0.44, *p* < 0.001; Expanded Numeracy Test—Subjective Numeracy Scale: *r*_(210)_ = 0.43, *p* < 0.001; Subjective Numeracy Scale—Berlin Advanced Numeracy Test: *r*_(212)_ = 0.35, *p* < 0.001], standardized measures. Cronbach's α for the composite measure was 0.67 and could not be increased by exclusion of any of the three separate measures^5^.

***Raven's Advanced Progressive Matrices (RAPM)***. Participants carried out a subset of Raven's progressive matrices (Raven et al., 1998) based on Stanovich and West (1998b). This test is generally used as a proxy to *fluid intelligence*. Participants were first instructed on the task. They were then allowed two of the 12 test items before completing 18 of the test items (items 13 through 30) with a 15 min time limit. Participants were instructed to try to complete all 18 items within the time limit.

***ANS—non-symbolic numerosity discrimination task***. On each of the 100 trials in the task based on Halberda et al. (2008) participants saw spatially intermixed blue and yellow dots on a monitor. Exposure time (200 ms) was too short for the dots to be serially counted. We used five ratios between the two sets of dots (1:2, 3:4, 5:6, 7:8, 9:10) with the total number of dots varying between 11 and 30. One fifth of the trials consisted of each ratio. For half of the trials, blue was the more numerous color, for the other half, yellow. Dots varied randomly in size. To counteract the use of perceptual cues we matched dot arrays either for total area or for average dot size. The participants judged which set was more numerous by pressing a color-coded keyboard button.

***Modeling of ANS acuity***. We used a classical psychophysics model that relies on a linear form of the ANS, to model performance in the ANS acuity task. Earlier work (e.g., Halberda et al., 2008) has shown this to be a plausible model of performance in numerical discrimination tasks. Percentage correct was modeled as a function of increasing ratio between the two sets of blue and yellow dots [larger sample (*n*_1_)/smaller sample (*n*_2_)]. The two sets are represented as Gaussian random variables with means *n*_1_ and *n*_2_ and standard deviations *w*· n_1_ and *w* · n_2_, respectively. Subtracting the Gaussian for the smaller set from that for the larger set returns a new Gaussian that has a mean of *n*_2_ − *n*_1_ and a standard deviation of $w\sqrt{n_{1}^{2}+n_{2}^{2}}$. Percentage correct is then equal to 1—error rate, where error rate is defined as the area under the tail of the resulting normal curve computed as follows

(2) $$\frac{1}{2}erfc\left(\frac{\left|n_{1}-n_{2}\right|}{\sqrt{2}w\sqrt{n_{1}^{2}+n_{2}^{2}}}\right),$$

where *erfc* is the complementary error function. This fits percentage correct in the ANS acuity task as a function of the Gaussian approximate number representation for the two sets of dots with *w* as a single free parameter. The individual Weber fraction obtained from such a model fit describes the standard deviations for the Gaussian representation of the ANS acuity, thus describing how much the two Gaussian representations overlap and thereby predicting an individual percentage correct on a numerical discrimination task. We used this model to find the best fit for each individual separately. All participants took part in the tasks above in the same order as follows; RAPM, ANS-task, Berlin advanced numeracy test, Expanded Numeracy test, Subjective numeracy scale, general knowledge task.

## Dependent measures

### Overconfidence and sources of miscalibration

In studies with general knowledge items, the participants are typically given a choice between two alternatives and have to indicate their confidence in this choice as a subjective probability in the interval 0.5 (guessing) and 1.0 (certain). For each participant, confidence ratings are obtained for a large number of items. The participants are said to be calibrated if in the long run the subjective probabilities are matched by the corresponding relative frequencies, that is, they have XX% correct answers in the confidence category with subjective probability.XX. The calibration score *C* is defined by the mean square deviation between confidence *x_t_* and the corresponding proportion correct *c_t_*,

(3) $$C=\frac{1}{N}\sum_{1}^{T}n_{t}{\left(x_{t}-c_{t}\right)}^{2}$$

where *n_t_* refers to the number of confidence judgments in confidence category *t* (*t* = 1..T), *N* refers to the overall number of confidence judgments, and *T* to the number of confidence categories available (see Lichtenstein et al., 1982). When the proportions correct equal the subjective probabilities at each confidence level, the participant is perfectly calibrated with a calibration score of 0. The over/underconfidence bias is measured by the difference between the mean confidence, *x*, and the overall proportion correct, *c*, where *x* − *c* > 0 indicates overconfidence and *x* − *c* < 0 is underconfidence. For instance, if the mean confidence is 0.8 but the overall proportion correct is 0.7, there is overconfidence 0.1. *Resolution* (Murphy, 1973) measures the ability of judges to distinguish incorrect from correct responses via confidence judgments. This variable is defined as the variance of proportion correct over confidence categories;

(4) $$R=\frac{1}{N}\sum_{1}^{T}n_{t}{\left(c_{t}-\bar{c}\right)}^{2}.$$

A high score of resolution reflects better performance than a low, with 0 as the worst possible value and the best value depends on the variance of proportion correct.

The calibration score C can be decomposed into three additive components (see Björkman, 1992);

(5) $$C=D^{2}+R^{2}+L$$

where *D* = *x* − *c*, *R*^2^ = *s_x_* − *s_c_* (the standard deviation of confidence minus the standard deviation of proportion correct) and L is 2*s_x_ s_c_* (1 − *r_xc_*) where *r_xc_* is the correlation between confidence and proportion correct. The first component, *D*^2^, measures the extent to which over/underconfidence contributes to poor calibration. The second component, *R*^2^, measures the degree to which poor discrimination between confidence categories contributes to poor calibration. *L*, or linearity, measures the degree to which the calibration curve is linear. That is, how much lack of linearity contributes to poor calibration. The linearity component is of particular interest in the following analyses given the discussion above about non-linearity in probability judgments.

#### Over/underestimation

Over/underestimation was measured by asking participants to estimate the number of questions that they believe they have answered correctly. The question was phrased: “In the previous task, how many out of the *x* (actual number) items do you think you answered correctly?” The measure of overestimation is the rated number of questions minus the observed number of questions, where a positive number indicated overconfidence (overestimation).

#### Overplacement

Overplacement was measured by asking participants to estimate the percentile rank of their performance (number of correctly answered questions in the general knowledge task they just had performed) relative to a random sample of participants with the characteristics of the actual comparison sample presented to them. The term percentile rank was carefully explained in a detailed way with examples in order for all participants to fully understand what they were rating. We used two measures of overplacement. The *numeric overplacement* measure is the actual percentile in performance on the general knowledge task subtracted from the estimated percentile, where a positive value indicates overconfidence. We also used a second *non-numeric overplacement* measure because even in spite of careful instructions, some participants may find the concept of percentiles hard to grasp. The non-numeric measure was constructed by asking participants to place their performance in one of four quartiles, described by the non-numeric labels “Definitely above the average,” “Slightly above the average,” “Slightly below the average,” and “Definitely below the average.” The measure was calculated as the numeric measure, by subtracting the actual quartile performance on the general knowledge task from the estimated quartile, where a positive value indicates overconfidence.

#### Conjunction fallacy

A conjunction fallacy occurs when the assessed probability of a conjunctive proposition *P*(*A*∩ B) is judged higher than at least one of the constituents (i.e., *P*(*A*) or *P*(*B*)).

## Results

Descriptive statistics for the dependent variables in the study are summarized in Table 1. In most regards, the data for this approximately population representative sample of citizens of Uppsala (save for the overrepresentation of women) are similar to the results from previous studies. For example, there is a minor overall overconfidence bias of 0.03, which is the standard finding when the proportion correct (0.67) for the item sample is slightly below the midpoint (0.75) of the confidence scale (Juslin et al., 2000). The participants moreover underestimate the number of correctly solved questions when asked for this number after responding to them (51 vs. 67, see Gigerenzer et al., 1991). In contrast to the common finding, the participants in this study underestimate their relative standing in the population (see “Placement”).

**Table 1:** **Means (m), Standard Deviations (s), and Number of Participants (N) for the dependent measures for the sample in the study (differing *N*s are due to missing data)**.

**Measure**	***m***	***s***	***N***
Numeracy (expanded test)	9.34	2.14	211
Numeracy (subjective)	3.93	0.98	212
Numeracy (BANT)	2.46	1.03	213
ANS (w)	0.25	0.09	213
RAPM	6.99	3.49	205
Prop. correct items	0.67	0.07	213
Confidence	0.70	0.09	213
Over/underconfidence bias	0.03	0.09	213
Calibration	0.02	0.02	213
Estimated correct questions (out of 100)	50.84	20.51	213
Proportion conjunction fallacies	0.50	0.15	211
Placement (non-numeric)	−0.02	0.29	213
Placement (numeric)	−0.07	0.28	213

Correlations between the main independent variables, dependent variables, and demographic variables in the entire sample can be found in Table 2. The correlation between ANS acuity measured by the weber fraction and the composite numeracy measure was low and non-significant [*r*_(212)_ = −0.10, *p* = 0.166]. ANS was significantly correlated with age, replicating the finding of a declining ANS acuity after 30 years of age (Halberda et al., 2012). Numeracy was slightly, but significantly, lower for women than for men. The correlation between Numeracy and the RAPM was strong, indicating that these measures overlap. RAPM correlated negatively with age, reproducing the well-known decline in fluid intelligence over the lifespan (e.g., Cavanaugh and Blanchard-Fields, 2006).

**Table 2:** **Zero-order correlations between the independent [1–5: Numeracy (NUM), ANS acuity (ANS), Ravens' Advanced Progressive Matrices (RAPM)] and Dependent Measures (6–13) included in the study**.

	**Measure**											
	**1**	**2**	**3**	**4**	**5**	**6**	**7**	**8**	**9**	**10**	**11**	**12**
1. Gender												
2. Age	−0.10											
3. RAPM	−0.06	−0.28^**^										
4. ANS	−0.07	0.24^**^	−0.13									
5. NUM	−0.14^*^	−0.07	0.46^*^	−0.10								
6. Over/underconfidence	−0.15^*^	−0.02	−0.23^*^	−0.02	−0.33^**^							
7. Calibration	−0.09	0.04	−0.22^*^	0.01	−0.33^**^	0.65^**^						
8. Resolution	−0.13	0.05	0.08	0.02	0.18^*^	0.02	−0.14^*^					
9. Linearity	−0.02	−0.11	−0.08	0.03	−0.32^**^	0.22^*^	0.47^**^	0.06				
10. Over/underestimation	−0.21^*^	0.09	0.09	−0.05	0.08	0.34^**^	0.18^*^	0.08	−0.04			
11. Overplacement (numeric)	0.07	−0.14^*^	−0.07	−0.02	−0.15^*^	0.50^**^	0.27^*^	0.03	0.09	0.31^**^		
12. Overplacement (non-numeric)	0.01	−0.12	−0.13	−0.03	−0.27^**^	0.57^**^	0.31^*^	−0.05	0.09	0.30^**^	0.83^**^	
13. Conjunction fallacy	−0.11	0.08	−0.14	−0.05	−0.21^*^	0.39^**^	0.17^**^	0.10	0.19^*^	0.24^**^	−0.06	0.05

*Gender is so that a positive point biserial correlation indicates higher values for women than for men. Lower values of ANS acuity indicates better performance*.

* *p < 0.05*,

** *p < 0.001*.

When not stated otherwise, the analyses below are performed as hierarchical multiple regression analyses with potentially confounding variables of gender, IQ (RAPM), proportion correct answers in the knowledge test^6^, and age entered at Stage 1 and the independent variables ANS acuity and Numeracy entered together at Stage 2. This was done to establish the independent contribution of the variable in question (ANS/numeracy). In the figures we will illustrate the main effect for the quartiles by using means adjusted for the potential contribution of these extraneous variables (both numeracy and ANS acuity were entered as continuous variables in the regressions). All full models are summarized in Table 3.

**Table 3:** **Summary of hierarchical regression analyses with control variable (gender, age, RAPM—Ravens' Advanced Progressive Matrices, and PC—Proportion Correct) inserted at Step 1 and numeracy and ANS acuity (w) inserted at Step 2**.

**Dependent variable**																
	**Over/under confidence**		**Calibration**		**Resolution**		**Linearity**		**Over/under estimation**		**Overplacement numeric**		**Overplacement non-numeric**		**Conjunction fallacy**	
**Predictor**	**Δ*****R***^**2**^	**β**	**Δ*****R***^**2**^	**β**	**Δ*****R***^**2**^	**β**	**Δ*****R***^**2**^	**β**	**Δ*****R***^**2**^	**β**	**Δ*****R***^**2**^	**β**	**Δ***R***^**2**^**	**β**	**Δ*R*^2^**	**β**
Step 1	0.24^**^		0.11^**^		0.02		0.04		0.06^*^		0.59^**^		0.53^**^		0.06^*^	−0.07
Gender		−0.25^**^		−0.12		−0.11		−0.04		−0.20^*^		−0.14^*^		−0.18^*^		0.01
Age		−0.01		0.05		−0.04		−0.11		0.11		−0.02		−0.02		−0.18^*^
RAPM		−0.14^*^		−0.14		−0.09		−0.09		0.12		0.13^*^		0.04		−17^*^
PC		−0.43^**^		−0.27^**^		0.004		−0.13		−0.04		−0.81^**^		−0.76^**^		
Step 2	0.04^*^		0.05^*^		0.02		0.09^*^		0.01		0.02^*^		0.02^*^		0.05^*^	
Numeracy		−0.21^*^		−0.26^*^		0.17^*^		−0.35^**^		0.05		0.1		−0.06		−0.26^*^
*w*		−0.11		−0.04		0.02		0.06		−0.09		−0.09^*^		−0.12^*^		−0.06
Total *R^2^*	0.28^**^		0.16^**^		0.04		0.13^**^		0.07^*^		0.61^*^		0.54^**^		0.11^**^	
*N*	205		205		205		205		205		205		205		203	

*Note: The table displays change in R^2^ (ΔR^2^) at each step, total R^2^ for the full model (Total R^2^), beta-weights for the predictors at each step (β), and the total number of participants included in each model (N)*.

* *p < 0.05*,

** *p < 0.001*.

### The effects of number knowledge on correspondence

IQ (RAPM), age, gender, and proportion of correctly solved items were entered in the first stage of a hierarchical multiple regression with calibration as the dependent variable. In the second stage, ANS acuity and Numeracy were entered. The potentially confounding variables entered at stage one contributed significantly to the regression model, [*F*_(4, 200)_ = 6.23, *p* < 0.001] and *R*^2^ = 0.11. Introducing the independent variables of main interest resulted in a significant change in *R*^2^, *F*_change_(2, 198) = 5.9, *p* = 0.003. Of these, the β-weight for ANS (−0.04) was not significant (*p* = 0.55) whereas the effect of Numeracy was (β = −0.26, *p* < 0.001). The effect of Numeracy and ANS on over/underconfidence was analyzed in a two stage hierarchical multiple regression as detailed above with over/underconfidence as dependent variable. Introducing the independent variables in the second stage resulted in a significant change in *R*^2^, *F*_change_(2, 198) = 5.75, *p* = 0.004. The β-weight for *w* (β = −0.108) failed to reach statistical significance (*p* = 0.086) but for Numeracy it was significant (β = −0.212, *p* = 0.003). For Murphy Resolution, the β-weight for Numeracy was significant (β = 0.17, *p* = 0.043), but the corresponding weight for *w* was non-significant (β = 0.02, *p* = 0.82). The linearity component (*L*) of the additive model of the calibration measure (Björkman, 1992) was used as a dependent variable in a hierarchical regression. Adding *w* and Numeracy in Step 2 significantly increased the *R*^2^ of the model [*F*_change_(2, 198) = 10.25, *p* < 0.001]. The weight for *w* was not significant (β = 0.06, *p* = 0.39), but the weight for numeracy was (β = −0.345, *p* < 0.001), indicating poorer linearity for those with lower numeracy.

**Figure 2:**
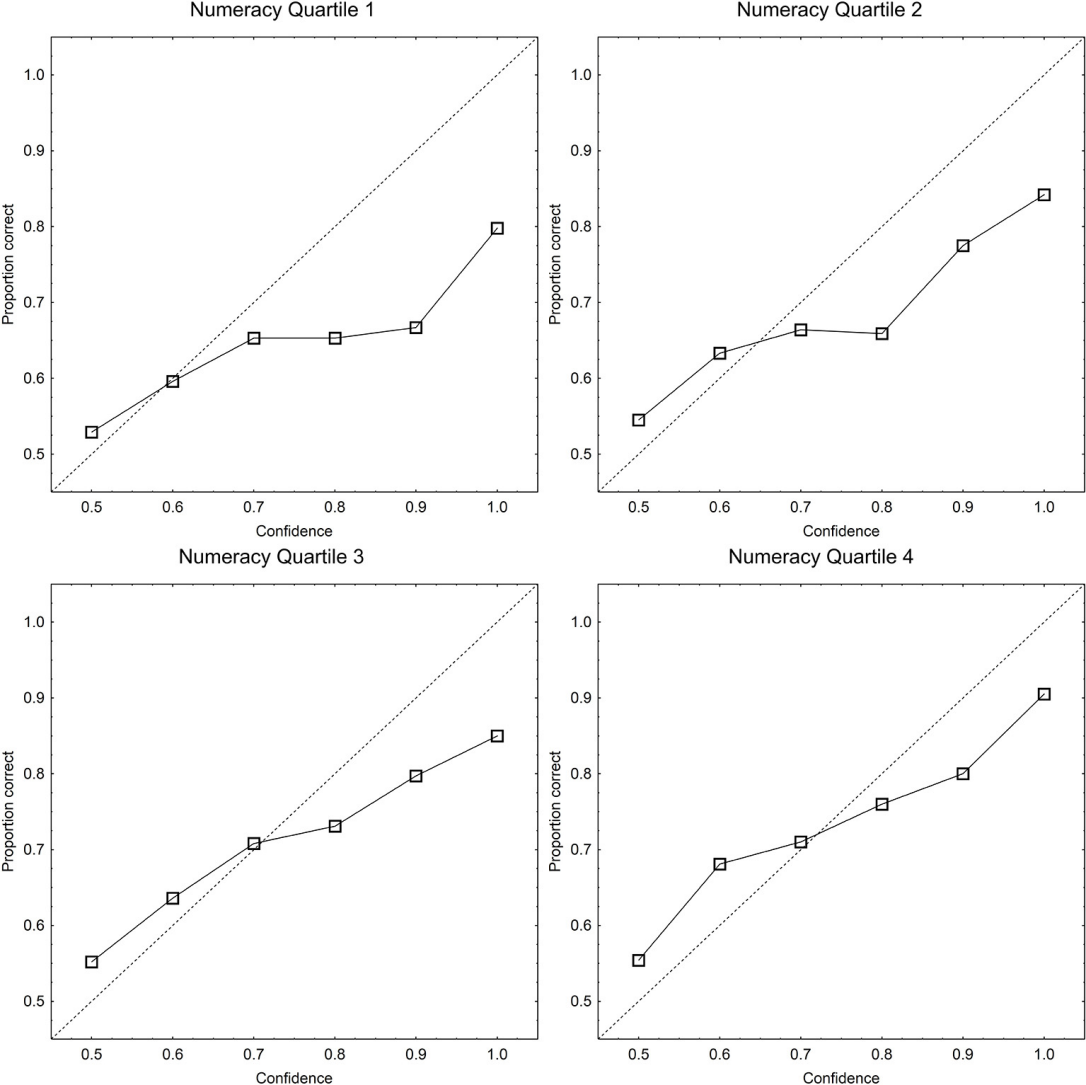
**Proportion correct plotted as function of confidence category (calibration curves) for the single statements in each of the numeracy quartiles**. The dotted identity line is perfect calibration.

**Figure 3:**
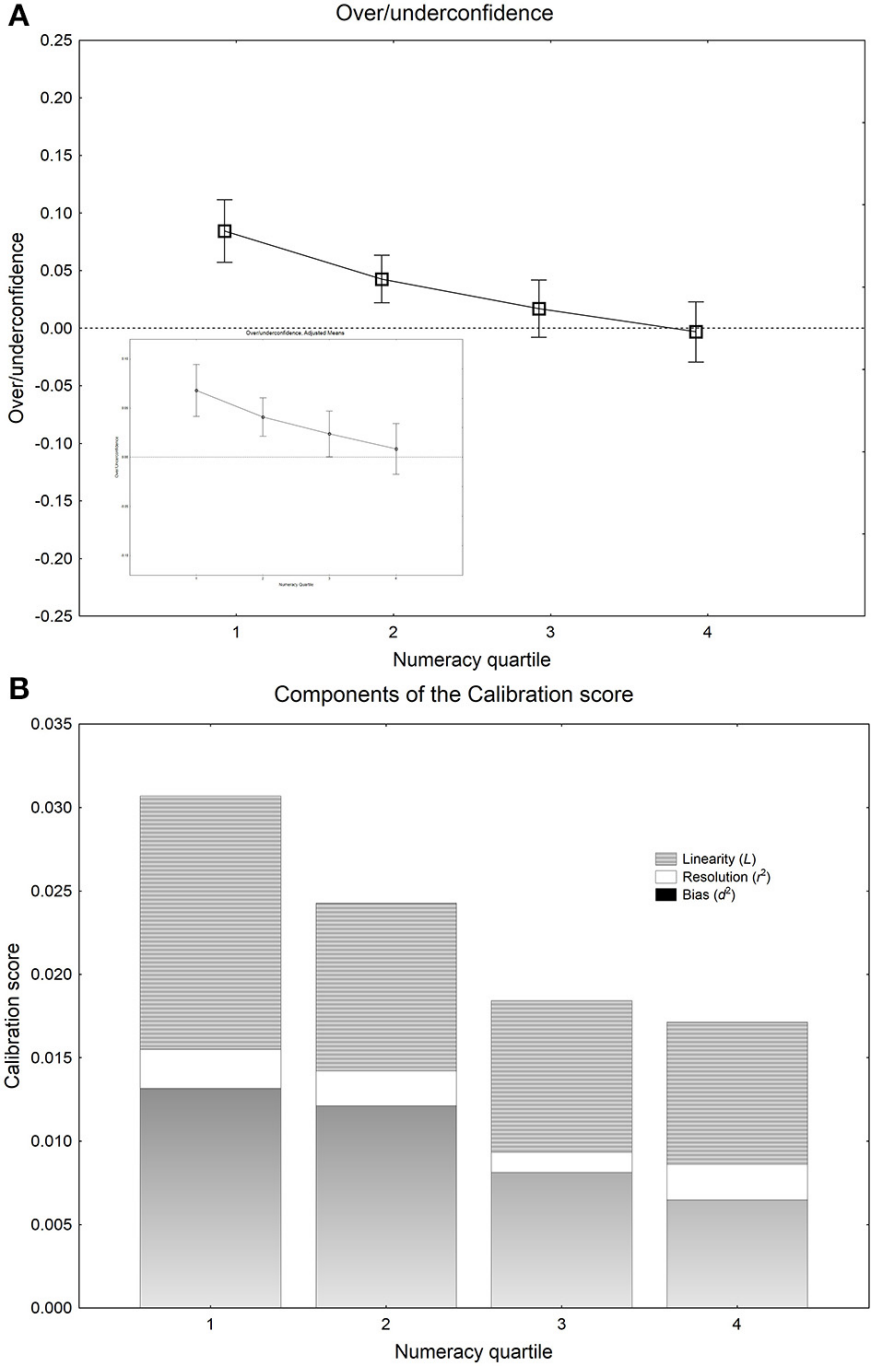
**(A)** The mean over/-underconfidence score with 95% confidence intervals plotted as function of the four numeracy quartiles. The dotted line is zero over/underconfidence. The small inserted panel shows the corresponding means with 95% confidence intervals, after adjustment for the effects of potentially confounding variables (see the main text). **(B)** The mean calibration score, where the areas indicate the portion of miscalibration contributed by each of the three additive components of the total Calibration score C (see Björkman, 1992).

The above analyses of calibration, overconfidence, resolution, and of the linearity component showed significant effects only of numeracy on the calibration of subjective probabilities. Because of this, a more detailed analysis involving calibration curves was undertaken with only this measure as an independent variable. Figure 2 shows calibration curves where proportions correct are plotted against confidence, for the single statements for participants in the four numeracy quartiles. The calibration curves are steeper and more linear for participants with higher numeracy than for participants with lower numeracy, which show curves indicative of overconfidence. The proportions correct in Figure 2 were subjected to a split-plot ANOVA, with confidence as within-subjects independent variable and numeracy quartile as between-subjects independent variable. There was a significant main effect of confidence [*F*_(5, 880)_ =137.61, *MSE* = 0.019, *p* < 0.001, partial η^2^ = 0.439] and of numeracy [*F*_(5, 880)_ = 12.4, *MSE* = 0.032, *p* < 0.001, partial η^2^ = 0.174], and a significant interaction [*F*_(5, 880)_ = 2.5, *MSE* = 0.019, *p* = 0.001, partial η^2^ = 0.041]. To probe the nature of the significant interaction with reference to our hypothesis we entered a contrast for steeper calibration curves at higher numeracy (crossing a linear trend for numeracy with a linear trend for confidence) and for more curvilinear calibration curves at higher numeracy (crossing a linear trend for numeracy with a quadratic trend for confidence). The calibration curves were significantly steeper at high numeracy [*F*_(1, 176)_ = 16.1, *MSE* = 0.015, *p* < 0.001] and significantly more nonlinear at lower numeracy [*F*_(1, 176)_ =3.99, *MSE* = 0.018, *p* = 0.047]^7^. As predicted if people with lower numeracy are less able to maintain a linear scale, lower numeracy is associated with less step and nonlinear calibration curves. The correlation between proportion correct and over/underconfidence was −0.41, although this is partially an artifact of the linear dependency between the measures (i.e., over/underconfidence bias is defined as mean confidence *minus proportion correct*; see Juslin et al., 2000). However, the pattern of overconfidence bias in the adjusted means was the same as in the raw means in Figure 3, with the 95% confidence intervals for the adjusted mean overconfidence deviating distinctly from 0 in numeracy quartiles 1 and 2, but not in quartiles 3 and 4 (see the inserted panel in Figure 3)^8^.

A measure of over/underestimation in the frequency estimates was calculated from estimates made of the number of correct answered questions in the general knowledge task. At all levels of numeracy, participants significantly underestimated the proportion of correctly solved items, but those with higher numeracy did so to a lesser extent. A single sample *t*-test shows that the mean (−0.16) significantly deviates from zero [*H*_0_ = 0, *t*_(212)_ = 12.45, *p* < 0.001]. Adding the independent variables at Step 2 did not contribute with improved statistical significance [*F*_change_(2, 198) = 1.04, = 0.35]. Neither the effect of *w* (β = −0.09, *p* = 0.21) nor the effect of Numeracy (β = 0.05, *p* = 0.51) was statistically significant.

**Figure 4:**
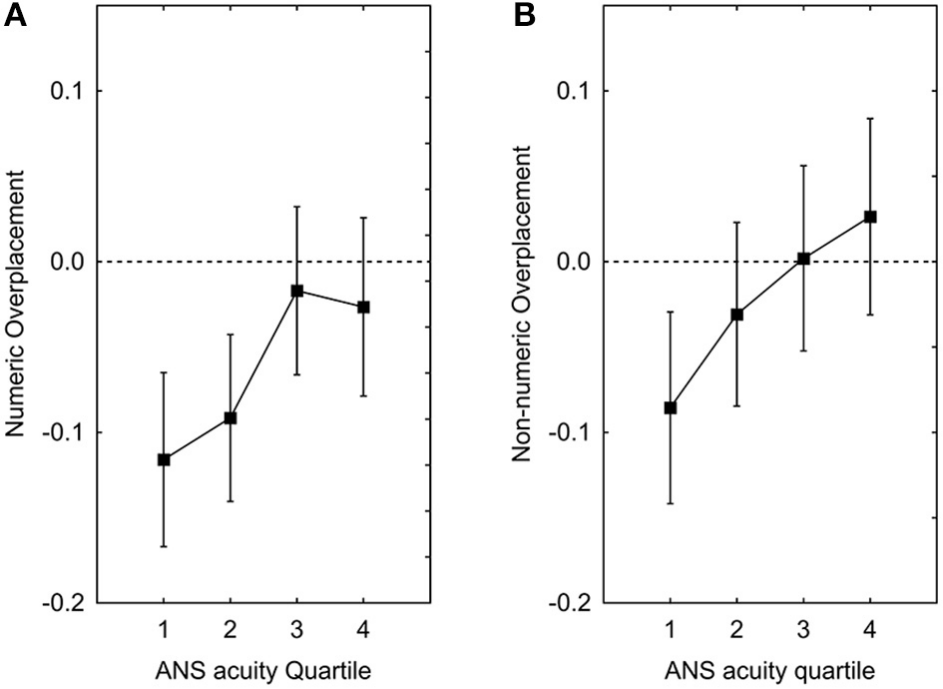
**Placement plotted as function of ANS Acuity Quartile for the numeric (A) and non-numeric measure (B)**. Square symbols depict means adjusted for the effects of potentially confounding variables (gender, age, IQ, and proportion of correct answers).

As for over/underplacement, participants underestimated their performance relative to others (average rated percentile = 43.5). The mean (−0.06) significantly deviates from zero [*H*_0_ = 0, *t*_(212)_ = 3.5, *p* < 0.001] for the numeric measure. For the non-numeric measure, the mean (−0.02) is slightly, but not to a statistically significant degree, different from zero [*H*_0_ = 0, *t*_(212)_ = 1.1, *p* = 0.26]. A hierarchical regression analysis with the non-numeric measure of over/underplacement as dependent variable showed that adding the independent variables at Step 2, did contribute with increased statistical significance [*F*_change_(2, 198) = 3.25, *p* = 0.041]. The effect of *w* was statistically significant (β = −0.12, *p* = 0.02) whereas the effect of Numeracy (β = −0.06, *p* = 0.29) was not significant. The corresponding analysis with the numeric measure of over/underplacement as dependent variable showed that adding the independent variables at Step 2, did contribute with increased statistical significance [*F*_change_(2, 198) = 3.8, *p* = 0.024]. The effect of *w* was statistically significant (β = −0.09, *p* = 0.04) whereas the effect of Numeracy (β = 0.09, *p* = 0.07) was not significant. As can be seen in Figures 4A,B, those with poorer ANS acuity underestimate their standing to a higher degree whereas those with more efficient ANS have a more realistic appreciation of their ability for both measures.

To summarize, the numeracy of the participants had significant effects on the calibration of subjective probabilities. Those higher in numeracy were generally better calibrated and obtained close to zero over/underconfidence bias in the judgments. By contrast, those lower in numeracy had much higher overconfidence and significantly larger deviations from a linear calibration curve. When metacognitive performance was measured as frequency estimates of correctly solved items, participants generally underestimated the proportion correctly solved items, but neither the effect of ANS acuity nor numeracy was statistically significant. When the metacognitive performance was measured by the relative standing compared to a defined population, again, participants underestimated their relative standing and there was only a significant effect for ANS acuity.

### The effect of number knowledge on coherence

A hierarchical regression analysis with the conjunction fallacy as dependent variable showed that adding ANS acuity and Numeracy at Step 2, did contribute with a statistical significance increase in *R^2^* [*F*_change_(2,196) = 5.7, *p* = 0.004]. The effect of *w* was not statistically significant (β = −0.06, *p* = 0.36) whereas the effect of Numeracy (β = −0.26, *p* < 0.001) was.

**Figure 5:**
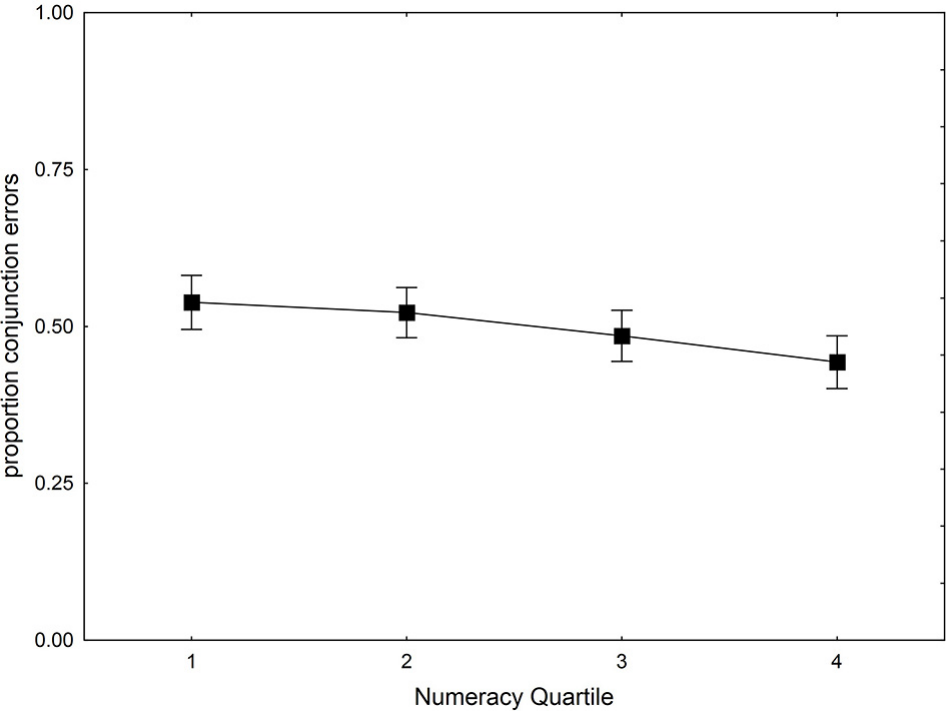
**Proportion of conjunction errors plotted as function of Numeracy Quartile**. Square symbols depict means adjusted for the effects of potentially confounding variables (gender, age, IQ, and proportion of correct answers).

In Figure 5, the proportion of conjunction errors is plotted as a function of numeracy quartile. The average participant committed 50% conjunction errors, which is close to the 47% observed by Nilsson et al. (2009) in a student-sample with an identical design. As is evident in Figure 5, the proportion of conjunction errors is similar in all numeracy quartiles, though slightly lower for participants higher in numeracy.

## Discussion

The present study contributes to the recent interest in how individual differences in numerical abilities are related to individual abilities in judgment and decision-making tasks in general (see e.g., Peters et al., 2008; Reyna et al., 2009; Liberali et al., 2012; Schley and Peters, 2014) and in particular to judgment tasks including probability judgments (e.g., Dieckmann et al., 2009; Lipkus et al., 2010). More specifically, the study examines how two separate (ANS acuity and Numeracy), but possibly related, numerical abilities relate to the correspondence and coherence of probability judgments. In addition, by using a representative design and a representative sample of participants we extended previous research concerned with the influence of individual differences on probability judgments.

### Numerical abilities and correspondence

Our measurements related to the correspondence criteria of rationality consisted of tasks with three different types of potential overconfidence; *miscalibration, overestimation*, and *overplacement* (see Merkle and Weber, 2011 for this taxonomy). As for miscalibration, direct assessments of confidence analyzed with subjective probability calibration were not related to ANS acuity but related to the numeracy of the participants. The participants with higher numeracy were better calibrated than those with lower numeracy. This suggests that numeracy taps abilities beyond those strictly required for rule-based analytic insights about probability, For example, the ability to maintain a linear numerical scale. Note also that this effect holds after controlling for proportion correct and RAPM. It is therefore not easily explained by differences between the groups in knowledge and general cognitive ability. Overconfidence in terms of miscalibration was statistically significant but small, as typically observed when the proportion correct is 0.67, and confined to the less numerate.

We predicted with our first hypothesis that the effect of numeracy on miscalibration might primarily be expressed as a higher degree of nonlinearity for less numerate participants. To investigate this possibility, we investigated the effect of numeracy on the linear component of the additive model of the calibration measure (Björkman, 1992). In accordance with our prediction, this investigation revealed that there was an effect on linearity (*L*), with more nonlinear calibration curves for less numerate participants. This result is consistent with previous research suggesting that performance in judgment and decision-making tasks is related to the appreciation of linearity (e.g., Schley and Peters, 2014). It is, however, important to notice that while numeracy was related to the degree of linearity, there was no effect of ANS acuity on the same measure. This goes against our second stated hypothesis. Schley and Peters (2014) found that a symbolic mapping measure, which is conceptually related to our ANS acuity measure, was associated with the shape of the value function but not with the shape of the weighting function. In that study, numeracy was significantly related the shape of the value function. The effect, however, went away when controlling for the symbolic mapping measure. Numeracy was not, however, related to the shape of the weighting function. Thus, in contrast to previous findings (e.g., Schley and Peters, 2014), our results suggest that the nonlinearity found in the representation of magnitudes and numbers might not be responsible for a similar nonlinearity in judgments and decisions. Instead, our results indicate that the culturally acquired ability to understand and manipulate exact numbers and/or perceptions of this ability and preference for numbers can contribute to linearity in judgments and decisions. It will be an important venue for future research to investigate the unique and combined contributions of culturally acquired and genetically predisposed abilities for understanding numerical information on nonlinearity in judgments and decisions.

There was no overestimation of the number of correctly solved items after completing the task. Instead there was strong underconfidence. This format dependence mimics the previously shown *confidence-frequency effect* (Gigerenzer et al., 1991; Schneider, 1995), but with a stronger underconfidence for frequency estimates. To a certain degree, this effect is boosted by the fact that some participants fail to appreciate that with a forced choice two alternative task there is a 50% success rate, and give very low estimates. This underestimation was neither related significantly to numeracy nor to ANS acuity.

The present study used a randomly sampled stimulus material, a locally recruited “quasi-random” sample of younger and middle-aged adult participants and well-defined performance. Under these conditions, we found no evidence indicating a “better than average effect” in our participants. That is, there was no indication of overplacement. In fact, with the numeric measure, participants generally believed they performed worse than average participant. Underplacement has been found in some studies with very easy tasks. Our task, however, had an intermediate level of difficulty (67% correct), so this cannot be the reason for our results. Benoît and Dubra (2011) criticized the entire paradigm of overplacement. They argued that even though a large number of studies show that people rate themselves as above average, this does not necessarily mean that people are overconfident. Instead, the finding of overplacement is fully compatible with a rational agent using Bayesian updating. Isolated findings (Merkle and Weber, 2011) have suggested that overplacement might occur even in designs robust to the critique by Benoît and Dubra (2011). However, in our own research we have investigated overplacement with other tasks (e.g., reasoning problems and numerosity judgments) and found very little support for a “better than average effect” with unambiguous skill definitions. Instead our data indicate a strong tendency for people to rate themselves as closer to the mean than they actually are.

The internal representation of magnitudes and numbers, indexed by the acuity of the ANS, has been found to be inherently nonlinear (Dehaene, 2009). In addition, previous research has indicated that judgments requiring processing of numerical information might be influenced by ANS acuity (Peters et al., 2008). We therefore hypothesized that ANS acuity and overconfidence could be negatively related. With respect to miscalibration and overestimation there was no effect of ANS acuity. It is possible that the difference in results to previous studies (Peters et al., 2008) is due to a difference in methodology. Peters et al. (2008) used a measure of ANS acuity (numeric distance effect) that been challenged for its validity (Lindskog et al., 2013; Chen and Li, 2014; Inglis and Gilmore, 2014). For example, Chen and Li (2014) found no correlation between the numeric distance effect and math performance in a meta-analysis, and both Inglis and Gilmore (2014) and Lindskog et al. (2013) report lack of positive associations between weber fractions and the numeric distance effect. We therefore used a measure that has been suggested to have better validity (Halberda et al., 2008; Lindskog et al., 2013). It is possible that the measure used by Peters et al. (2008) taps a different construct than the measure used here and that that construct is somehow related to judgments requiring processing of numerical information. Another possibility is that the relationship between ANS acuity and such judgments is too elusive to be consistently captured, or that the varying results may be due to low reliability of ANS measures^9^. Future research will need to resolve this question.

The acuity of the ANS was, however, related to the measures of over/underplacement. What is the reason for this effect? A tentative, but admittedly *post hoc* explanation, may be that the metacognitive ability involved in this task explicitly draws on magnitude ordering in locating the order of one's own performance relative to others. It has been shown that both Rhesus monkeys (Cantlon and Brannon, 2006) and 11-month-old children (Brannon, 2002) have the ability to discriminate between magnitudes in terms of relative order. This could mean that ordinality is a basic property of the ANS. Lyons and Beilock (2011) showed that symbolic number-ordering ability fully mediates the observed relation between approximate number acuity and mental arithmetic. They proposed that this indicates that relative ordering may be a stepping stone from approximate number representation to mathematical competence. The neurological underpinnings of such a system were recently identified by Knops and Willmes (2013) who showed that corresponding areas in the intra parietal sulcus and in the inferior frontal cortex were activated similarly when participants were involved in rank order judgments and mental arithmetic. More research is clearly needed to replicate and extend these findings. In the mean time we propose that ANS acuity influences metacognitive ability due to its basic function as the building block of rank order judgments.

### Numerical abilities and coherence

The coherence of participants' probability judgments was evaluated by the extent to which they made the conjunction fallacy. We predicted with our third hypothesis that people of higher numeracy would be more likely to adhere to the extension rule of probability theory and thus to commit fewer conjunction errors. In line with this prediction we found that the rate of conjunction fallacies was reduced by numeracy. However, unlike overconfidence in subjective probability calibration, even those of higher numeracy still committed a high degree of conjunction errors. The results on coherence thus suggested that the rate of conjunction fallacies is not strongly mediated by the kind of analytical insight captured by numeracy. Rather, if anything, they derive from more general computation constraints that are not as easily amended by culturally acquired knowledge of the analytical rules of probability (Juslin et al., 2009, 2011; Nilsson et al., 2009).

Again, the results show (e.g., Nilsson et al., 2009) that it is not necessary for a researcher to come up with imaginative scenarios involving feminist bank clerks named Linda to observe the conjunction fallacy. Conjunction fallacies will appear as frequently in scenarios where there is no dependency between the conjuncts, and when there is no sensible possibility at all for people to rely on a “representativeness heuristic.” The apparent robustness of this phenomenon to differences in stimulus material is another piece of evidence as to the phenomenon's roots in more general psychological mechanisms.

The lack of previous research linking ANS acuity to judgment and decision-making tasks made strong predictions about a possible relationship difficult. However, previous research indicating a relationship between ANS acuity and math achievement (e.g., Halberda et al., 2008; Lindskog et al., 2013) has suggested, if anything, that people with better ANS acuity would be more inclined to appreciate the conjunction rule. Our results indicated no effect of ANS acuity on the prevalence of conjunction errors. We have previously proposed that although ANS acuity is related to performance in basic arithmetic tasks, such as addition and subtraction, the relationship might not extend to more advanced mathematics (Lindskog et al., 2013, 2014). That we failed to find and effect of ANS acuity might therefore indicate that appreciation of the conjunction rule requires computational skills and conceptual understanding that goes beyond basic arithmetic.

## Conclusions

In the present study we investigated how individual abilities in understanding numerical information relates to probability judgments. We extended previous research on how individual differences influence probability judgments by using a sample of younger and middle-aged adults recruited outside university campus and by using a representative design. In general, our results suggest that the culturally acquired ability of numeracy and preference for numbers is related to both the coherence and the correspondence of probability judgments. More specifically, people higher on numeracy tend to be both more coherent in their probability judgments and to give probability judgments that are more in correspondence with the world than do people lower on numeracy. We also found evidence that an innate ability to understand magnitudes and numbers, captured by the ANS, was related to one metacognitive type of judgment. Those with better ANS acuity gave more realistic estimates of their performance relative to others.

Measures that have traditionally been taken as relatively general and unproblematic such as for example overconfidence, conjunction fallacy and risk aversion may have to be reconsidered. These measures, that have been viewed as tapping into a content-independent rationality and associated judgment biases, may in fact be confounded with a relatively specific and newly culturally acquired skill involving the understanding and use of numbers. This need not imply that these biases are any less important in a world that makes increasing quantitative demands on human cognition, but it suggests that one may need to exercise some caution in generalizing behaviors observed in these numerical tasks to behaviors in contexts less contingent on understanding and using numbers. It may not primarily be the nature of the slow and explicit cognitive processes in System II, approximated by Numeracy in the present study, that are a remedy to the biases, but rather the conceptual understanding of the content conveyed by these processes. In the present study, however, even participants high in numeracy were highly susceptible to the conjunction fallacy. This suggests that System II might not be the default mode of operation, even to the tutored mind.

### Conflict of interest statement

The authors declare that the research was conducted in the absence of any commercial or financial relationships that could be construed as a potential conflict of interest.

## Footnotes

^1^Here we are dealing with the perception of a stated proportion or percentage *per se* and we will not distinguish between the case where the proportion refers to a single event probability with a stochastic component or to a relative frequency in a reference class, which need not have a stochastic component if no random sampling is applied to the reference class. Therefore, we will use the terms “proportion” and “probability” interchangeably in the following. From other points of view there may although be important differences between them.

^2^Equation 1 only takes the slope parameter *k* and not the intercept parameter *p0* into account, because in the data reported below the intercept parameter is constrained to equal 0.5 by use of half-range probability assessments on the interval 0.5–1. For the general version of the model, see Zhang and Maloney (2012).

^3^Liberali et al. (2012) showed that objective and subjective numeracy tests measure constructs that overlap, but are not identical. We chose to use a composite measure that will tap numeracy in a broader sense, tapping both of these constructs. Results for each separate tests were, however similar to those presented here.

^4^A comprehensive list of all tasks can be obtained from the authors.

^5^Because of the fixed order in which the numeracy tests were performed, it is possible that results on the Subjective Numeracy Scale were colored by participants' performance on the other numeracy tests. However, in a previous study (Lindskog et al., submitted) with a Latin Square balanced order we found overall comparable correlations [Expanded-Subjective *r*_(119)_ = 0.47, *p* < 0.001, Berlin-Subjective *r*_(119)_ = 0.41, *p* < 0.001]. The correlations with Subjective Numeracy and the other measures were also obtained when this test was taken prior to the other numeracy tests [Expanded-Subjective: *r*_(36)_ = 0.35, *p* = 0.03; Berlin-Subjective: *r*_(36)_ = 0.36, *p* = 0.03]. Thus, whereas the correlation between subjective numeracy and the other measures may have been somewhat boosted by the fixed order in the present study, the correlation *per* se is not an artifact due to this order.

^6^Because of the possible linear dependency between proportion correct and the various dependent measure (see e.g., Juslin et al., 2000), this variable was used as a potentially confounding variable. For example, overconfidence is the difference between proportion correct and mean confidence. As a consequence, those performing at poor levels may look overconfident due to mere regression effects or scale-end effects. We wanted to avoid this possibility and investigate metacognitive effects independent of performance level. As a consequence, the resulting analyses will reveal more pure effects, but probably at the cost of being quite conservative.

^7^The corresponding contrast analysis of the main effect of confidence revealed a significant linear trend [*F*_(1, 176)_ = 877.189, *MSE* = 0.015, *p* < 0.001], but no significant quadratic (nonlinear) trend [*F*_(1, 176)_ = 1.470, *MSE* = 0.018, *p* = 0.227]. For the numeracy quartile there was likewise a significant linear trend [*F*_(1, 176)_ = 37.051, *MSE* = 0.015, *p* < 0.001], but no quadratic (nonlinear) trend [*F*_(1, 176)_ = 0.233, *MSE* = 0.018, *p* = 0.630].

^8^It is of, course, also possible to compute the overconfidence bias across the conjunctive statements. We performed repeated measures ANOVA with numeracy quartile as the independent variable and overconfidence for single statements and for conjunctive statements as dependent variables. There was a significant main effect of single or conjunctive statement [*F*_(1, 206)_ = 71.383, *MSE* = 0.005, *p* < 0.001, *partial* η^2^ = 0.257], with more overconfidence for the conjunctive than for the single statements (overall means of 0.096 vs. 0.0.035). There was also a significant main effect of numeracy quartile [*F*_(3, 206)_ = 8.205, *MSE* = 0.018, *p* < 0.001, *partial* η^2^ = 0.107], with more overconfidence for the participants with low rather than high numeracy. There was no significant interaction between statement and numeracy [*F*_(3, 206)_ < 1, *MSE* = 0.005, *p* < 0.543, *partial* η^2^ = 0.010]. In short: there was more overconfidence with the conjunctive statements, but the same effect of numeracy.

^9^We have no exact figure of the reliability of the ANS test used in the present sample. However, we have reason to suspect reliability for the typical test in general to be modest (Lindskog et al., 2013) both for numerosity discriminations, and the symbolic numeric distance effect. For example, Lindskog et al. estimated a mere reliability of 0.4 for weber fractions obtained in the Halberda et al. (2008) task [Halberda et al. (2012) reported reliabilities of 0.56 and 0.72 in two tasks with the same stimuli].
